# Complete convergence and complete moment convergence for weighted sums of extended negatively dependent random variables under sub-linear expectation

**DOI:** 10.1186/s13660-017-1538-1

**Published:** 2017-10-23

**Authors:** Haoyuan Zhong, Qunying Wu

**Affiliations:** College of Science, Guilin University of Technology, Guilin, 541004 P.R. China

**Keywords:** 60F15, sub-linear expectation space, END random variables, complete convergence, complete moment convergence

## Abstract

In this paper, we study the complete convergence and complete moment convergence for weighted sums of extended negatively dependent (END) random variables under sub-linear expectations space with the condition of $C_{\mathbb{V}}[|X|^{p}l(|X|^{1/\alpha})]<\infty$, further $\hat{\mathbb {E}}(|X|^{p}l(|X|^{1/\alpha}))\leq C_{\mathbb{V}}[|X|^{p}l(|X|^{1/\alpha })]<\infty$, $1< p<2$ ($l(x)>0$ is a slow varying and monotone nondecreasing function). As an application, the Baum-Katz type result for weighted sums of extended negatively dependent random variables is established under sub-linear expectations space. The results obtained in the article are the extensions of the complete convergence and complete moment convergence under classical linear expectation space.

## Introduction

Additivity has been generally regarded as a fairly natural assumption, so the classical probability theorems have always been considered under additive probabilities and the linear expectations. However, many uncertain phenomena do not satisfy this assumption. So Peng [1–5] introduced the notions of sub-linear expectations to extend the classical linear expectations. He also established the general theoretical framework of the sub-linear expectation space. The theorems of sub-linear expectations are widely used to assess financial riskiness under uncertainty. For complete convergence and complete moment convergence, there are few reports under sub-linear expectations. This paper aims to obtain the complete convergence and complete moment convergence under sub-linear expectation space with the condition of $C_{\mathbb{V}}[|X|^{p}l(|X|^{1/\alpha})]<\infty$, further $\hat{\mathbb{E}}(|X|^{p}l(|X|^{1/\alpha}))\leq C_{\mathbb{V}}[|X|^{p}l(|X|^{1/\alpha})]<\infty$, $1< p<2$. In addition, the results and conditions of this paper include a slow varying and monotone nondecreasing function, so the theorems are more generic than the traditional complete convergence. In a word, it is meaningful that this paper extends the complete convergence and complete moment convergence under sub-linear expectation.

Sub-linear expectations generate lots of interesting properties which are unlike those in linear expectations, and the issues in sub-linear expectations are more challenging, so lots of scholars have attached importance to them. Numbers of results have been established, for example, Peng [1–5] gained a weak law of large numbers and a central limit theorem under sub-linear expectation space. Chen [6] gained the law of large numbers for independent identically distributed random variables with the condition of $\hat{\mathbb{E}}(|X|^{1+\alpha})<\infty $. The powerful tools as the moment inequalities and Kolmogorov’s exponential inequalities were established by Zhang [7–9]. He also obtained the Hartman-Wintner’s law of iterated logarithm and Kolmogorov’s strong law of large numbers for identically distributed and extended negatively dependent random variables. Wu and Chen [10] also researched the law of the iterated logarithm, and Cheng [11] studied the strong law of larger number with a general moment condition $\sup_{i\geq1}\hat{\mathbb{E}}[|X_{i}|\psi(|X_{i}|)]<\infty$, and so on. Many powerful inequations and conventional methods for linear expectation and probabilities are no longer valid, the study of limit theorems under sub-linear expectation becomes much more challenging.

The complete convergence has a relatively complete development in probability limit theory. The notion of complete convergence was raised by Hsu and Robbins [12], and Chow [13] established complete moment convergence. The complete moment convergence is a more general version of the complete convergence. Lots of results on complete convergence and complete moment convergence for different sequences have been found under classical probability space. For example, Shen *et al*. [14], Wang *et al*. [15] and Wu and Jiang [16], and so on. Some recent papers had new results about complete convergence and complete moment convergence. For instance, Wang *et al*. [17] gained general results of complete convergence and complete moment convergence for weighted sums of some class of random variables, and Wang *et al*. [18] researched complete convergence and complete moment convergence for a class of random variables, and so on. In addition, the theorems of this paper are the extensions of the literature [14] under sub-linear expectation space. And we prove the theorems in this paper with the condition of $C_{\mathbb{V}}[|X|^{p}l(|X|^{1/\alpha})]<\infty$, further $\hat{\mathbb {E}}(|X|^{p}l(|X|^{1/\alpha}))\leq C_{\mathbb{V}}[|X|^{p}l(|X|^{1/\alpha })]<\infty$, $1< p<2$ ($l(x)>0$ is a slow varying function).

In the next section, we generally introduce some basic notations and concepts, related properties under sub-linear expectations and preliminary lemmas that are useful to prove the main theorems. In Section 3, the complete convergence and complete moment convergence under sub-linear expectation space are established. The proofs of these theorems are stated in the last section.

## Basic settings

The study of this paper uses the framework and notations which are established by Peng [1–5]. So, we omit the definitions of sub-linear expectation ($\hat{\mathbb{E}}$), capacity $(\mathbb{V},v)$, countably sub-additive and Choquet integrals/expectations $(C_{\mathbb{V}},C_{v})$ and so on.

### Definition 2.1

(Peng [1, 2], Zhang [8])

(i) (Identical distribution) Assume that a space $\textbf{X}_{1}$ and a space $\textbf{X}_{2}$ are two *n*-dimensional random vectors defined severally in the sub-linear expectation space $(\Omega_{1},\mathcal {H}_{1},\hat{\mathbb{E}}_{1})$ and $(\Omega_{2},\mathcal{H}_{2},\hat{\mathbb {E}}_{2})$. They are named identically distributed if
  $$ \hat{\mathbb{E}}_{1}\bigl[\varphi(\textbf{{X}}_{1}) \bigr]=\hat{\mathbb {E}}_{2}\bigl[\varphi(\textbf{{X}}_{2}) \bigr],\quad\forall\varphi\in C_{l,\mathrm{Lip}}(\mathbb{R}_{n}), $$
  whenever the sub-expectations are finite. A sequence $\{X_{n}, n\geq1\}$ of random variables is named to be identically distributed if, for each $i\geq1,X_{i}$ and $X_{1}$ are identically distributed.
(ii) (Extended negatively dependent) A sequence of random variables $\{ X_{n}, n\geq1\}$ is named to be upper (resp. lower) extended negatively dependent if there is some dominating constant $K\geq1$ such that
  $$ \hat{\mathbb{E}} \Biggl(\prod_{i=1}^{n}g_{i}(X_{i}) \Biggr)\leq K\prod_{i=1}^{n}\hat{\mathbb{E}} \bigl(g_{i}(X_{i})\bigr), \quad\forall n\geq2. $$
  Whenever the nonnegative functions $g_{i}(X_{i})\in C_{b,\mathrm{Lip}}(\mathbb{R})$, $i=1,2,\ldots $ , are all nondecreasing (resp. all nonincreasing). They are named extended negatively dependent if they are both upper extended negatively dependent and lower extended negatively dependent.

It is distinct that if $\{X_{n}, n\geq1\}$ is a sequence of extended independent random variables and $f_{1}(x),f_{2}(x),\ldots{}\in C_{l,\mathrm{Lip}}(\mathbb{R})$, then $\{f_{n}(X_{n}), n\geq1\}$ is also a sequence of extended dependent random variables with $K=1$; if $\{X_{n}, n\geq1\} $ is a sequence of upper extended negatively dependent random variables and $f_{1}(x),f_{2}(x),\ldots{}\in C_{l,\mathrm{Lip}}(\mathbb{R})$ are all nondecreasing (resp. all nonincreasing) functions, then $\{f_{n}(X_{n});n\geq 1\}$ is also a sequence of upper (resp. lower) extended negatively dependent random variables. It shall be noted that the extended negative dependence of $\{X_{n}, n\geq1\}$ under $\hat{\mathbb{E}}$ does not imply the extended negative dependence under *ε̂*.

In the following, let $\{X_{n}, n\geq1\}$ be a sequence of random variables in $(\Omega,\mathcal{H},\hat{\mathbb{E}})$ and $\sum_{i=1}^{n}X_{i}=S_{n}$. The symbol *C* is on behalf of a generic positive constant which may differ from one place to another. Let $a_{n}\ll b_{n}$ denote that there exists a constant $C>0$ such that $a_{n}\leq Cb_{n}$ for sufficiently large *n*, $I(\cdot)$ denotes an indicator function, $a_{x}\sim b_{x}$ denotes $\lim_{x\rightarrow\infty}\frac{a_{x}}{b_{x}}=1$. Also, let $a_{n}\approx b_{n}$ denote that there exist constants $c_{1}>0$ and $c_{2}>0$ such that $c_{1}a_{n}\leq b_{n}\leq c_{2}a_{n}$ for sufficiently large *n*.

The following three lemmas are needed in the proofs of our theorems.

### Lemma 2.1

([19])

$l(x)$ *is a slow varying function if and only if*

2.1 $$ l(x)=c(x)\operatorname{exp} \biggl\{ \int_{1}^{x}\frac{f(u)}{u} \,\mathrm{d}u \biggr\} ,\quad x>0, $$

*where* $c(x)\geq0$, $\lim_{x\rightarrow\infty}c(x)=c>0$, *and* $\lim_{x\rightarrow\infty}f(x)=0$.

### Lemma 2.2

*Suppose* $X\in\mathcal{H}$, $p>0$, $\alpha>0$, *and* $l(x)$ *is a slow varying function*.

(i) *Then*, *for* $\forall c>0$,
  2.2 $$ C_{\mathbb{V}}\bigl[|X|^{p}l\bigl(|X|^{1/\alpha}\bigr)\bigr]< \infty\quad\Leftrightarrow\quad\sum_{n=1}^{\infty}n^{\alpha p-1}l(n)\mathbb{V}\bigl(|X|>c n^{\alpha}\bigr)< \infty. $$
(ii) *If* $C_{\mathbb{V}}[|X|^{p}l(|X|^{1/\alpha})]<\infty$, *then for any* $\theta>1$ *and* $c>0$,
  2.3 $$ \sum_{k=1}^{\infty}\theta^{k\alpha p}l\bigl( \theta^{k}\bigr)\mathbb{V}\bigl(|X|>c\theta ^{k\alpha}\bigr) < \infty. $$

### Proof

(i) By Lemma 2.1, we can express $l(x)$ as equality (2.1), and $f(u)\rightarrow0$ as $u\rightarrow\infty$, $c(x)\rightarrow c$ as $x\rightarrow\infty$. Let $Z(x)=|x|^{p}l(|x|^{1/\alpha})$, $Z^{-1}(x)$ be the inverse function of $Z(x)$, $l(x)$ is a slow varying function and for any $c>0$, we have

$$\begin{aligned} C_{\mathbb{V}}\bigl[|X|^{p}l \bigl(|X|^{1/\alpha}\bigr)\bigr]&= \int_{0}^{\infty}\mathbb {V} \bigl(|X|^{p}l \bigl(|X|^{1/\alpha}\bigr)>x \bigr)\,\text{d}x \\ &= \int_{0}^{\infty}\mathbb{V} \bigl(|X|>Z^{-1}(x):=c y^{\alpha}\bigr)\,\text{d}x \\ &= \int_{0}^{\infty}\mathbb{V} \bigl(|X|>c y^{\alpha}\bigr) \bigl(c\alpha py^{\alpha p-1}l(cy)+y^{\alpha p-1}l(cy)cf(y)\bigr) \,\text{d}y \\ &\sim \int_{0}^{\infty}\mathbb{V} \bigl(|X|>c y^{\alpha}\bigr)\alpha p y^{\alpha p-1}l(y)\,\text{d}y.\end{aligned} $$

So,

$$ C_{\mathbb{V}}\bigl[|X|^{p}l\bigl(|X|^{1/\alpha} \bigr)\bigr] < \infty \quad\Leftrightarrow\quad\sum_{n=1}^{\infty}n^{\alpha p-1}l(n)\mathbb{V}\bigl(|X|>c n^{\alpha}\bigr)< \infty. $$

(ii) By the proof of (i), we can imply that for any $\theta>1$

$$ \begin{aligned}\infty &> \sum_{n=1}^{\infty}n^{\alpha p-1}l(n)\mathbb{V}\bigl(|X|>C n^{\alpha}\bigr)\\ &\geq C \sum _{k=1}^{\infty}\sum_{\theta^{k-1}\leq n< \theta ^{k}} \theta^{k(\alpha p-1)}l\bigl(\theta^{k}\bigr)\mathbb{V}\bigl(|X|>C \theta^{k\alpha}\bigr) \\ &\approx\sum_{k=1}^{\infty}\theta^{k\alpha p}l\bigl(\theta^{k}\bigr)\mathbb {V}\bigl(|X|>C \theta^{k\alpha}\bigr).\end{aligned} $$

 □

### Lemma 2.3

(Zhang [9] (Rosenthal’s inequalities))

*Let* $\{ X_{n},n\geq1\}$ *be a sequence of upper extended negatively dependent random variables in* $(\Omega,\mathcal{H},\hat{\mathbb{E}})$. *And* $\hat {\mathbb{E}}[X_{k}]\leq0$, $k=1,\ldots,n$. *Then*

2.4 $$ \mathbb{V}(S_{n}\geq x)\leq(1+Ke)\frac{\sum_{k=1}^{n}\hat{\mathbb {E}}(X_{k})^{2}}{x^{2}},\quad\forall x\geq0. $$

## Main results

### Theorem 3.1

*Let* $0< p<2$, $\alpha>0$, $\alpha p>1$, *and* $\{X_{n},n\geq1\} $ *be a sequence of END and identically distributed random variables under sub*-*linear expectations*. *Let* $l(x)>0$ *be a slow varying and monotone nondecreasing function*. *And* $\{a_{ni},1\leq i\leq n,n\geq1\}$ *is an array of real numbers such that*

3.1 $$ \sum_{i=1}^{n} a_{ni}^{2} =O(n). $$

*If*

3.2 $$ C_{\mathbb{V}}\bigl[|X|^{p}l\bigl(|X|^{1/\alpha} \bigr)\bigr]< \infty, $$

*further*, *for* $1< p<2$,

3.3 $$ \hat{\mathbb{E}}\bigl(|X|^{p}l\bigl(|X|^{1/\alpha}\bigr)\bigr)\leq C_{\mathbb{V}}\bigl[|X|^{p}l\bigl(|X|^{1/\alpha}\bigr)\bigr]. $$

*Then*, *for any* $\varepsilon>0$,

3.4 $$ \sum_{n=1}^{\infty}n^{\alpha p-2}l(n) \mathbb{V} \Biggl(\sum_{i=1}^{n}a_{ni}(X_{i}-b_{i})> \varepsilon n^{\alpha}\Biggr)< \infty, $$

*where* $b_{i}=0$ *if* $p\leq1$, *and* $b_{i}=\hat{\mathbb{E}}X_{i}$ *if* $p>1$;

3.5 $$ \sum_{n=1}^{\infty}n^{\alpha p-2}l(n) \mathbb{V} \Biggl(\sum_{i=1}^{n}a_{ni}(X_{i}-b_{i})< - \varepsilon n^{\alpha}\Biggr)< \infty, $$

*where* $b_{i}=0$ *if* $p\leq1$, *and* $b_{i}=\hat{\mathbb{\varepsilon}}X_{i}$ *if* $p>1$.

In particular, if $\hat{\mathbb{E}}X_{i}=\hat{\varepsilon}X_{i}$, then

3.6 $$ \sum_{n=1}^{\infty}n^{\alpha p-2}l(n) \mathbb{V} \Biggl( \Biggl\vert \sum_{i=1}^{n}a_{ni}(X_{i}-b_{i}) \Biggr\vert >\varepsilon n^{\alpha}\Biggr)< \infty, $$

where $b_{i}=0$ if $p\leq1$, and $b_{i}=\hat{\mathbb{E}}X_{i}=\hat{\mathbb {\varepsilon}}X_{i}$ if $p>1$.

### Theorem 3.2

*Suppose that the conditions of Theorem* 3.1 *hold*, *and* $\hat{\mathbb{E}}X_{i}=\hat{\mathbb{\varepsilon}}X_{i}=b_{i}$, ${1< p<2}$, *then*, *for any* $\varepsilon>0$,

3.7 $$ \sum_{n=1}^{\infty}n^{\alpha p-2-\alpha}l(n)C_{\mathbb{V}} \Biggl[ \Biggl\vert \sum_{i=1}^{n}a_{ni}(X_{i}-b_{i}) \Biggr\vert -\varepsilon n^{\alpha}\Biggr]^{+}< \infty. $$

### Theorem 3.3

*Suppose that* $1/2<\alpha\leq1$ *and other conditions of Theorem* 3.1 *hold*. *Let* ${l(x)>0}$ *be a monotone nondecreasing function*. *Assume further that* $\{a_{ni},1\leq i\leq n,n\geq1\}$ *is an array of real numbers such that* (3.1) *holds and* $\hat {\mathbb{E}}X_{i}=\hat{\mathbb{\varepsilon}}X_{i}=b_{i}$. *If*

3.8 $$ \hat{\mathbb{E}}\bigl(|X|^{1/\alpha}l\bigl(|X|^{1/\alpha}\bigr)\bigr)\leq C_{\mathbb{V}}\bigl[|X|^{1/\alpha}l\bigl(|X|^{1/\alpha}\bigr)\bigr]< \infty, $$

*then*, *for* $\forall\varepsilon>0$,

3.9 $$ \sum_{n=1}^{\infty}\frac{l(n)}{n}\mathbb{V} \Biggl(\sum_{i=1}^{n}a_{ni}(X_{i}-b_{i})> \varepsilon n^{\alpha}\Biggr)< \infty. $$

## Proof

### Proof of Theorem 3.1

Without loss of generality, we can assume that $\hat{\mathbb{E}}X_{i}=0$, when $p>1$. We just need to prove (3.5). Because of considering $\{-X_{n};n\geq1\}$ instead of $\{X_{n};n\geq1\} $ in (3.5), we can obtain (3.6). Noting that $a_{ni}\geq0$, without loss of generality, we can assume that

4.1 $$ \sum_{i=1}^{n}a_{ni}^{2} \leq Cn, $$

and $a_{ni}\geq0$ for all $1\leq i\leq n$ and $n\geq1$. It follows by (3.2) and Hölder’s inequality that

4.2 $$ \sum_{i=1}^{n}a_{ni}\leq \Biggl(n \sum_{i=1}^{n}a_{ni}^{2} \Biggr)^{1/2}\leq Cn. $$

For fixed $n\geq1$, denote for $1\leq i\leq n$ that

$$\begin{gathered} X_{i}^{(n)}=-n^{\alpha}I\bigl(X_{i}< -n^{\alpha}\bigr)+X_{i}I\bigl(|X_{i}|\leq n^{\alpha}\bigr)+n^{\alpha}I\bigl(X_{i}>n^{\alpha}\bigr), \\T^{(n)}=n^{-\alpha}\sum_{i=1}^{k}a_{ni} \bigl(X_{i}^{(n)}-\hat{\mathbb{E}}X_{i}^{(n)} \bigr).\end{gathered} $$

It is easily checked that for $\forall\varepsilon>0$,

$$ \Biggl(\sum_{i=1}^{n}a_{ni}X_{i}> \varepsilon n^{\alpha}\Biggr)\subset\bigcup_{i=1}^{n} \bigl(|X_{i}|> n^{\alpha}\bigr)\cup \Biggl(\sum _{i=1}^{n}a_{ni}X_{i}^{(n)}> \varepsilon n^{\alpha}\Biggr), $$

which can imply that

$$\begin{gathered} \sum_{n=1}^{\infty}n^{\alpha p-2}l(n)\mathbb{V} \Biggl(\sum_{i=1}^{n}a_{ni}X_{i}> \varepsilon n^{\alpha}\Biggr) \\ \quad\leq\sum_{n=1}^{\infty}n^{\alpha p-2}l(n)\sum_{i=1}^{n} \mathbb {V}\bigl(|X_{i}|> n^{\alpha}\bigr) \\ \qquad{} +\sum_{n=1}^{\infty}n^{\alpha p-2}l(n)\mathbb{V} \Biggl(T^{(n)}>\varepsilon-\Bigg|n^{-\alpha} \sum_{i=1}^{n}a_{ni}\hat{\mathbb {E}}X_{i}^{(n)}\Bigg| \Biggr) \\ \quad:= I_{1}+I_{2}.\end{gathered} $$

For $0<\mu<1$, let $g(x)$ be a decreasing function and $g(x)\in C_{l,\mathrm{Lip}}(\mathbb{R})$, $0\leq g(x)\leq1$ for all *x* and $g(x)=1$ if $|x|\leq\mu$, $g(x)=0$ if $|x|>1$. Then

4.3 $$ I\bigl(|x|\leq\mu\bigr)\leq g(x)\leq I\bigl(|x|\leq1\bigr),\qquad I\bigl(|x|\geq1\bigr)\leq1- g(x)\leq I\bigl(|x|\geq \mu\bigr). $$

In order to prove (3.5), it suffices to show $I_{1}<\infty$ and $I_{2}<\infty $. By Lemma 2.2(i) and identically distributed random variables, we can get that

$$\begin{aligned} I_{1}&\leq C\sum_{n=1}^{\infty}n^{\alpha p-2}l(n)\sum_{i=1}^{n}\hat { \mathbb{E}} \biggl(1-g \biggl(\frac{X_{i}}{n^{\alpha}} \biggr) \biggr) \\ &\leq C\sum_{n=1}^{\infty}n^{\alpha p-1}l(n) \hat{\mathbb{E}} \biggl(1-g \biggl(\frac{X}{n^{\alpha}} \biggr) \biggr) \\ &\leq C\sum_{n=1}^{\infty}n^{\alpha p-1}l(n)\mathbb{V}\bigl(|X|>\mu n^{\alpha}\bigr)< \infty.\end{aligned} $$

In the following, we prove that $I_{2}<\infty$. First, we prove that

4.4 $$ \Biggl\vert n^{-\alpha}\sum_{i=1}^{n}a_{ni} \hat{\mathbb{E}}X_{i}^{(n)} \Biggr\vert \rightarrow0 \quad \text{as } n\rightarrow\infty. $$

Case 1: $0< p\leq1$.

For any $r>0$, by the $C_{r}$ inequality and (4.4),

4.5 $$\begin{aligned}& \big|X^{(n)}\big|^{r}\ll|X|^{r}I\bigl(|X|\leq n^{\alpha}\bigr)+n^{r\alpha}I\bigl(|X|> n^{\alpha}\bigr)\leq |X|^{r}g \biggl(\frac{\mu X}{n^{\alpha}} \biggr)+n^{r\alpha} \biggl(1-g \biggl(\frac{ X}{n^{\alpha}}\biggr) \biggr), \\& \begin{aligned}[b]\hat{\mathbb{E}}\big|X^{(n)}\big|^{r} &\ll \hat{ \mathbb{E}} \biggl[|X|^{r}g \biggl(\frac{\mu X}{n^{\alpha}} \biggr) \biggr]+n^{r\alpha}\hat {\mathbb{E}} \biggl[1-g\biggl(\frac{ X}{n^{\alpha}} \biggr) \biggr] \\ &\leq \hat{ \mathbb{E}} \biggl[|X|^{r}g \biggl(\frac{\mu X}{n^{\alpha}} \biggr) \biggr]+n^{r\alpha}\mathbb{V}\bigl(|X|>\mu n^{\alpha}\bigr).\end{aligned} \end{aligned}$$

So, by (4.3) we can imply that

4.6 $$ \begin{aligned}[b] \Biggl\vert n^{-\alpha}\sum_{i=1}^{n}a_{ni} \hat{\mathbb {E}}X_{i}^{(n)} \Biggr\vert &\ll n^{-\alpha}\hat{\mathbb{E}} \bigl\vert X^{(n)} \bigr\vert \sum _{i=1}^{n}a_{ni} \\ &\leq n^{1-\alpha}\hat{\mathbb{E}} \bigl\vert X^{(n)} \bigr\vert \\ &\leq Cn^{1-\alpha} \biggl(\hat{\mathbb{E}}|X|g \biggl( \frac{\mu X}{n^{\alpha}} \biggr)+n^{\alpha}\mathbb{V} \bigl(|X|>\mu n^{\alpha}\bigr) \biggr) \\ &\leq Cn^{1-\alpha}\hat{\mathbb{E}}|X|g \biggl(\frac{\mu X}{n^{\alpha}} \biggr)+Cn\mathbb{V}\bigl(|X|>\mu n^{\alpha}\bigr) \\ &:= I_{21}+Cn\mathbb{V}\bigl(|X|>\mu n^{\alpha}\bigr).\end{aligned} $$

By (2.3), we can imply that

$$ \infty>\sum_{n=1}^{\infty}n^{\alpha p-1}l(n) \mathbb{V} \bigl(|X|>cn^{\alpha}\bigr)\geq\sum _{n=1}^{\infty}\mathbb{V} \bigl(|X|>cn^{\alpha}\bigr), $$

and $\mathbb{V}(|X|>\mu n^{\alpha})\downarrow$, so we get $n\mathbb {V}(|X|>\mu n^{\alpha})\rightarrow0$ as $n\rightarrow\infty$. Next, we estimate $I_{21}$. Let $g_{j}(x)\in C_{l,\mathrm{Lip}}(\mathbb{R})$, $j\geq1$ such that $0\leq g_{j}(x)\leq1$ for all *x* and $g_{j}(\frac{x}{2^{j\alpha}})=1$ if $2^{(j-1)\alpha}<|x|\leq2^{j\alpha}$, $g_{j}(\frac{x}{2^{j\alpha}})=0$ if $|x|\leq2^{(j-1)\alpha}$ or $|x|>(1+\mu)2^{j\alpha}$. Then

4.7 $$ g_{j} \biggl(\frac{X}{2^{j\alpha}} \biggr)\leq I \bigl( \mu2^{(j-1)\alpha }< |X|\leq(1+\mu)2^{j\alpha} \bigr), \qquad X^{r}g \biggl(\frac{X}{2^{k\alpha }} \biggr)\leq1+\sum_{j=1}^{k}X^{r}g_{j} \biggl(\frac{X}{2^{j\alpha}} \biggr). $$

For every *n*, there exists *k* such that $2^{k-1}\leq n<2^{k}$, thus by (4.7), $g(x)\downarrow$, and $n^{-\alpha+1}\downarrow0$, from $\alpha >\frac{1}{p}\geq1$, we get

$$\begin{aligned} I_{21} &\leq 2^{(k-1)(1-\alpha)}\hat{\mathbb{E}}|X|g \biggl( \frac {\mu X}{2^{k\alpha}} \biggr) \\ &\leq C2^{(k-1)(1-\alpha)}\sum_{j=1}^{k} \hat{\mathbb {E}}|X|g_{j} \biggl(\frac{\mu X}{2^{j\alpha}} \biggr) \\ &\leq 2^{(k-1)(1-\alpha)}\sum_{j=1}^{k}2^{j\alpha} \mathbb{V} \bigl(|X|>2^{(j-1)\alpha} \bigr).\end{aligned} $$

Noting that by (2.4), $\alpha p>1$,

$$\begin{aligned} \sum_{j=1}^{\infty}\frac{2^{j\alpha}}{2^{j(\alpha-1)}}\mathbb {V}\bigl(|X|>2^{(j-1)\alpha}\bigr)&= \sum _{j=1}^{\infty}2^{j}\mathbb {V} \bigl(|X|>2^{-\alpha}2^{j\alpha}\bigr) \\ &\leq \sum_{j=1}^{\infty}2^{j\alpha p}l \bigl(2^{j}\bigr)\mathbb{V}\bigl(|X|>2^{-\alpha }2^{j\alpha} \bigr)< \infty.\end{aligned} $$

It follows that

$$ I_{21}\rightarrow0 \quad\text{as } n\rightarrow\infty $$

from the Kronecker lemma and $2^{j(\alpha-1)}\uparrow\infty$.

Case 2: $1< p<2$.

By (3.4), we can get that

4.8 $$ \hat{\mathbb{E}}|X|^{p}< \infty. $$

By (4.9) and $\alpha p>1$, $1< p<2$, one can get that

$$\begin{aligned} \Biggl\vert n^{-\alpha}\sum_{i=1}^{n}a_{ni} \hat{\mathbb {E}}X_{i}^{(n)} \Biggr\vert &\leq n^{-\alpha}\sum_{i=1}^{n}a_{ni} \bigl\vert \hat{\mathbb{E}}X_{i}-\hat {\mathbb{E}}X_{i}^{(n)} \bigr\vert \\ &\leq n^{-\alpha}\sum_{i=1}^{n}a_{ni} \hat{\mathbb {E}}\big|X_{i}-X_{i}^{(n)}\big| \\ &\leq n^{1-\alpha}\frac{\hat{\mathbb{E}}|X||X|^{p-1}}{n^{\alpha (p-1)}} \biggl(1-g \biggl( \frac{X}{n^{\alpha}} \biggr) \biggr) \\ &\ll Cn^{1-\alpha p}\hat{\mathbb{E}}|X|^{p}\rightarrow0 \quad \text{as }n\rightarrow\infty.\end{aligned} $$

It follows that for all *n* large enough,

$$ \Bigg|n^{-\alpha}\sum_{i=1}^{n}a_{ni} \hat{\mathbb{E}}X_{i}^{(n)}\Bigg|< \varepsilon/2, $$

which implies that

$$ I_{2}\leq C \sum_{n=1}^{\infty}n^{\alpha p-2}l(n)\mathbb{V} \bigl(T^{(n)}>\varepsilon/2 \bigr). $$

By Definition 2.1(ii), we can know that fixed $n\geq1,\{ a_{ni}(X_{i}^{(n)}-\hat{\mathbb{E}}X_{i}^{(n)}),1\leq i\leq n\}$ are still END random variables. Hence, we have by Lemma 2.3 (taking $x=\varepsilon n^{\alpha}$) that

$$\begin{aligned} \mathbb{V}\bigl(T^{(n)}>\varepsilon/2\bigr)&\leq C \frac{\sum_{i=1}^{n}\hat {\mathbb{E}} (a_{ni}(X_{i}^{(n)}-\hat{E}X_{i}^{(n)}) )^{2}}{\varepsilon^{2}n^{2\alpha}} \\ &\leq C n^{-2\alpha}\sum_{i=1}^{n}a_{ni}^{2} \hat{\mathbb{E}}\bigl(X_{i}^{(n)}\bigr)^{2}.\end{aligned} $$

By (4.6), we have

$$\begin{aligned} I_{2} \leq {}& C \sum_{n=1}^{\infty}n^{\alpha p-2\alpha-2}l(n)\sum_{i=1}^{n} a_{ni}^{2}\hat{\mathbb{E}} \bigl\vert X_{i}^{(n)} \bigr\vert ^{2} \\ \leq{}& C\sum_{n=1}^{\infty}n^{\alpha p-2\alpha-1}l(n)\hat{\mathbb {E}} \biggl[X^{2}g \biggl( \frac{\mu X}{n^{\alpha}} \biggr) \biggr]\\ &+C\sum_{n=1}^{\infty}n^{\alpha p-1}l(n)\mathbb{V} \bigl(|X|>\mu n^{\alpha}\bigr) \\ :={}& I_{3}+I_{4}.\end{aligned} $$

By Lemma 2.2(i), we can get $I_{4}<\infty$. Noting that by (4.8)

$$\begin{aligned} I_{3}& = C \sum_{j=0}^{\infty}\sum_{n=2^{j}}^{2^{j+1}-1}n^{\alpha p-2\alpha-1}l(n)\hat{ \mathbb{E}} \biggl[X^{2}g \biggl(\frac{\mu X}{n^{\alpha}} \biggr) \biggr] \\ &\leq C \sum_{j=1}^{\infty}2^{(\alpha p-2\alpha-1)j}2^{j}l \bigl(2^{j}\bigr)\hat {\mathbb{E}} \biggl[X^{2}g \biggl( \frac{\mu X}{2^{\alpha(j+1)}} \biggr) \biggr] \\ &\leq C\sum_{j=1}^{\infty}2^{\alpha(p-2)j}l \bigl(2^{j}\bigr)\hat{\mathbb {E}} \Biggl[1+\sum _{k=1}^{j}X^{2}g_{k} \biggl( \frac{\mu X}{2^{\alpha(k+1)}} \biggr) \Biggr] \\ &\leq C\sum_{j=1}^{\infty}2^{\alpha(p-2)j}l \bigl(2^{j}\bigr)+C\sum_{j=1}^{\infty}2^{\alpha(p-2)j}l \bigl(2^{j}\bigr)\sum_{k=1}^{j} \hat{\mathbb{E}} \biggl[X^{2}g_{k} \biggl(\frac{\mu X}{2^{\alpha(k+1)}} \biggr) \biggr] \\ &= I_{31}+I_{32}.\end{aligned} $$

By $p<2$, we get $I_{31}<\infty$. Next we estimate $I_{32}$. By (2.4), we can imply that

$$\begin{aligned} I_{32} &= \sum_{j=1}^{\infty}2^{\alpha(p-2)j}l \bigl(2^{j}\bigr)\sum_{k=1}^{j} \hat{\mathbb{E}} \biggl[X^{2}g_{k} \biggl(\frac{\mu X}{2^{\alpha (k+1)}} \biggr) \biggr] \\ &\leq \sum_{k=1}^{\infty}2^{\alpha pk}l \bigl(2^{k}\bigr)\hat{\mathbb{E}} \biggl[g_{k} \biggl( \frac{\mu X}{2^{\alpha(k+1)}} \biggr) \biggr] \\ &\leq \sum_{k=1}^{\infty}2^{\alpha pk}l \bigl(2^{k}\bigr)\mathbb{V}\bigl(|X|>2^{\alpha k}\bigr)\\ &< \infty.\end{aligned} $$

Hence, it follows that

$$ I_{3}< \infty. $$

By $I_{3}<\infty$ and $I_{4}<\infty$, we can get $I_{2}<\infty$.

This finishes the proof of Theorem 3.1. □

### Proof of Theorem 3.2

Without loss of generality, we can assume that $\hat{\mathbb{E}}X_{i}=0$ when $p>1$, and assume that $a_{ni}\geq0$. For $\forall\varepsilon>0$, we have by Theorem 3.1 that

$$\begin{gathered} \sum_{n=1}^{\infty}n^{\alpha p-2-\alpha}l(n)C_{\mathbb{V}} \Biggl(\sum_{i=1}^{n}a_{ni}(X_{i}-b_{i})- \varepsilon n^{\alpha}\Biggr)^{+} \\ \quad =\sum_{n=1}^{\infty}n^{\alpha p-2-\alpha}l(n) \int_{0}^{\infty}\mathbb{ V} \Biggl(\sum _{i=1}^{n}a_{ni}X_{i}-\varepsilon n^{\alpha}>t \Biggr)\,\text{d}t \\ \quad = \sum_{n=1}^{\infty}n^{\alpha p-2-\alpha}l(n) \int_{0}^{n^{\alpha}} \mathbb{ V} \Biggl(\sum _{i=1}^{n}a_{ni}X_{i}-\varepsilon n^{\alpha}>t \Biggr)\,\text{d}t \\ \quad \quad{} +\sum_{n=1}^{\infty}n^{\alpha p-2-\alpha}l(n) \int_{n^{\alpha}}^{\infty}\mathbb{V} \Biggl(\sum _{i=1}^{n}a_{ni}X_{i}-\varepsilon n^{\alpha}>t \Biggr)\,\text{d}t \\ \quad \leq\sum_{n=1}^{\infty}n^{\alpha p-2}l(n) \mathbb{V} \Biggl(\sum_{i=1}^{n}a_{ni}X_{i}> \varepsilon n^{\alpha}\Biggr) \\ \quad \quad{} +\sum_{n=1}^{\infty}n^{\alpha p-2-\alpha}l(n) \int_{n^{\alpha}}^{\infty}\mathbb{ V} \Biggl(\sum _{i=1}^{n}a_{ni}X_{i}-\varepsilon n^{\alpha}>t \Biggr)\,\text{d}t \\ \quad \leq C\sum_{n=1}^{\infty}n^{\alpha p-2-\alpha}l(n) \int_{n^{\alpha}}^{\infty}\mathbb{V} \Biggl(\sum _{i=1}^{n}a_{ni}X_{i}>t \Biggr) \,\text{d}t.\end{gathered} $$

Hence, it suffices to show that

$$ H:=\sum_{n=1}^{\infty}n^{\alpha p-2-\alpha}l(n) \int_{n^{\alpha}}^{\infty}\mathbb{V}\Biggl(\sum _{i=1}^{n}a_{ni}X_{i}>t\Biggr) \,\text{d}t< \infty. $$

For $t>n^{\alpha}$, denote

4.9 $$ Z_{ti}=-tI(X_{i}< -t)+X_{i}I\bigl(|X_{i}| \leq t\bigr)+tI(X_{i}>t),\quad i=1,2,\ldots $$

and

4.10 $$ U_{ti}=tI(X_{i}< -t)+X_{i}I\bigl(|X_{i}|>t\bigr)-tI(X_{i}>t),\quad i=1,2, \ldots. $$

Since $X_{i}=U_{ti}+Z_{ti}$, it follows that

$$\begin{aligned} H \leq{}& \sum_{n=1}^{\infty}n^{\alpha p-2-\alpha}l(n) \int _{n^{\alpha}}^{\infty}\mathbb{V}\Biggl(\sum _{i=1}^{n}a_{ni}X_{i}>t\Biggr) \,\text{d}t \\ \leq{}& \sum_{n=1}^{\infty}n^{\alpha p-2-\alpha}l(n) \int_{n^{\alpha}}^{\infty}\mathbb{V}\Biggl(\Bigg|\sum _{i=1}^{n}a_{ni}U_{ti}\Bigg|>t/2\Biggr) \,\text{d}t \\ &+ \sum_{n=1}^{\infty}n^{\alpha p-2-\alpha}l(n) \int_{n^{\alpha}}^{\infty}\mathbb{V} \Biggl(t^{-1}\sum _{i=1}^{n}a_{ni}(Z_{ti}- \hat{\mathbb {E}}Z_{ti})>1/2-t^{-1}\Bigg|\sum _{i=1}^{n}a_{ni}\hat{\mathbb{E}}Z_{ti}\Bigg| \Biggr)\,\text{d}t \\ :={}&H_{1}+H_{2}.\end{aligned} $$

Note that by Lemma 2.2(i)

4.11 $$ \begin{aligned}[b] H_{1} &\leq C\sum_{n=1}^{\infty}n^{\alpha p-2-\alpha}l(n) \int _{n^{\alpha}}^{\infty}\mathbb{V} \bigl(\exists1\leq i< n, |X_{i}|>t \bigr)\,\text{d}t \\ &\leq C\sum_{n=1}^{\infty}n^{\alpha p-2-\alpha}l(n) \int _{n^{\alpha}}^{\infty}\sum_{i=1}^{n} \mathbb{V}\bigl(|X_{i}|>t\bigr)\,\text{d}t \\ &\leq C\sum_{n=1}^{\infty}n^{\alpha p-1-\alpha}l(n) \int _{n^{\alpha}}^{\infty}\hat{\mathbb{E}} \biggl( 1-g \biggl( \frac{X}{t} \biggr) \biggr)\,\text{d}t \\ &= C\sum_{n=1}^{\infty}n^{\alpha p-1-\alpha}l(n)\sum_{m=n}^{\infty}\int_{m^{\alpha}}^{(m+1)^{\alpha}}\hat{\mathbb{E}} \biggl( 1-g \biggl( \frac{X}{t} \biggr) \biggr)\,\text{d}t \\ &\leq C\sum_{n=1}^{\infty}n^{\alpha p-1-\alpha}l(n)\sum_{m=n}^{\infty}\bigl[(m+1)^{\alpha}-m^{\alpha} \bigr]\hat{\mathbb {E}} \biggl( 1-g \biggl(\frac{X}{m^{\alpha}} \biggr) \biggr) \\ &\leq C\sum_{m=1}^{\infty}m^{\alpha-1}\mathbb{V}\bigl(|X|>\mu m^{\alpha}\bigr)\sum _{n=1}^{m} n^{\alpha p-1-\alpha}l(n) \\ &\ll \sum_{m=1}^{\infty}m^{\alpha p-1}l(m) \mathbb{V}\bigl(|X|>\mu m^{\alpha}\bigr)< \infty.\end{aligned} $$

In the following, we prove that $H_{2}<\infty$. First, we show that

4.12 $$ \sup_{t\geq n^{\alpha}}t^{-1}\Bigg|\sum_{i=1}^{n}a_{ni} \hat{\mathbb {E}}Z_{ti}\Bigg|\rightarrow0 \quad\text{as } n\rightarrow\infty. $$

Case 1: $0< p\leq1$.

Note (4.10) and (4.4), which imply that

4.13 $$\begin{aligned}& |Z_{ti}|\ll|X_{i}|I\bigl(|X_{i}|\leq t\bigr)+tI\bigl(|X_{i}|>t\bigr)\leq|X_{i}|g \biggl(\frac{\mu X_{i}}{t} \biggr)+t \biggl(1-g \biggl(\frac{ X_{i}}{t} \biggr) \biggr), \\& \begin{aligned}[b] \hat{\mathbb{E}}|Z_{ti}| &\ll\hat{ \mathbb{E}} \biggl[|X|g \biggl(\frac{\mu X}{t} \biggr) \biggr]+t\hat{\mathbb{E}} \biggl[1-g \biggl(\frac{ X}{t} \biggr) \biggr] \\ &\leq \hat{ \mathbb{E}} \biggl[|X|g \biggl(\frac{\mu X}{t} \biggr) \biggr]+t \mathbb{V}\bigl(|X|>\mu t\bigr). \end{aligned} \end{aligned}$$

So, for $t>n^{\alpha}$, we get

$$\begin{aligned} \sup_{t\geq n^{\alpha}}t^{-1} \Biggl\vert \sum _{i=1}^{n}a_{ni}\hat{\mathbb {E}}Z_{ti} \Biggr\vert &\ll \sup_{t\geq n^{\alpha}}t^{-1}n \hat{\mathbb{E}}|Z_{ti}| \\ &\leq \sup_{t\geq n^{\alpha}}t^{-1}n \biggl(\hat{\mathbb {E}}|X|g \biggl(\frac{\mu X}{t} \biggr)+t\mathbb{V}\bigl(|X|>\mu t\bigr) \biggr) \\ &\leq n^{1-\alpha}\hat{\mathbb{E}}|X|g \biggl(\frac{\mu X}{n^{\alpha}} \biggr)+n\mathbb{V}\bigl(|X|>\mu n^{\alpha}\bigr) \\ &:= H_{21}+n\mathbb{V}\bigl(|X|>\mu n^{\alpha}\bigr).\end{aligned} $$

We get $n\mathbb{V}(|X|>\mu n^{\alpha})\rightarrow0$ as $n\rightarrow \infty$ in the proof of (4.7). Next, we estimate $H_{21}$. For every *n*, there exists *k* such that $2^{k-1}\leq n<2^{k}$, thus by (4.8), (4.13), $g(x)\downarrow$, $t>n^{\alpha}$ and $n^{-\alpha+1}\downarrow0$, from $\alpha>1$, we get

$$\begin{aligned} H_{21} &\leq Cn^{1-\alpha}\hat{\mathbb{E}}|X|g \biggl( \frac{\mu X}{n^{\alpha}} \biggr) \\ &\leq 2^{(k-1)(1-\alpha)}\hat{\mathbb{E}}|X|g \biggl(\frac{\mu X}{2^{k\alpha}} \biggr) \\ &\leq 2^{(k-1)(1-\alpha)}\sum_{j=1}^{k} \hat{\mathbb{E}}|X|g \biggl(\frac{\mu X}{2^{k\alpha}} \biggr) \\ &\leq 2^{(k-1)(1-\alpha)}\sum_{j=1}^{k}2^{j\alpha} \mathbb {V}\bigl(|X|>2^{(j-1)\alpha}\bigr).\end{aligned} $$

Noting that by (2.4), $\alpha p>1$,

$$\begin{aligned} \sum_{j=1}^{\infty}\frac{2^{j\alpha}}{2^{j(\alpha-1)}}\mathbb {V}\bigl(|X|>2^{(j-1)\alpha}\bigr)&= \sum _{j=1}^{\infty}2^{j}\mathbb {V} \bigl(|X|>2^{-\alpha}2^{j\alpha}\bigr) \\ &\leq \sum_{j=1}^{\infty}2^{j\alpha p}l \bigl(2^{j}\bigr)\mathbb{V}\bigl(|X|>2^{-\alpha }2^{j\alpha} \bigr)< \infty.\end{aligned} $$

It follows that

$$ H_{21}\rightarrow0 \quad\text{as } n\rightarrow\infty $$

from the Kronecker lemma and $2^{j(\alpha-1)}\uparrow\infty$.

Case 2: $1< p<2$.

By $\hat{\mathbb{E}}X_{i}=0$ and $\alpha p>1$, $t>n^{\alpha}$, we can get that

$$\begin{aligned} \sup_{t\geq n^{\alpha}} t^{-1} \Biggl\vert \sum _{i=1}^{n}a_{ni}\hat{\mathbb {E}}Z_{ti} \Biggr\vert &\leq \sup_{t\geq n^{\alpha}} t^{-1}\sum_{i=1}^{n}a_{ni} \vert \hat{\mathbb {E}}X_{i}-\hat{\mathbb{E}}Z_{ti} \vert \\ &\leq n^{-\alpha}\sum_{i=1}^{n}a_{ni} \hat{\mathbb{E}} \bigl\vert X_{i}-X_{i}^{(n)} \bigr\vert \\ &\leq Cn^{1-\alpha}\frac{\hat{\mathbb {E}}|X||X|^{p-1}}{n^{\alpha(p-1)}} \biggl(1-g \biggl( \frac{X}{n^{\alpha}} \biggr) \biggr) \\ &= Cn^{1-\alpha p}\hat{\mathbb{E}}|X|^{p} \biggl(1-g \biggl( \frac{X}{n^{\alpha}} \biggr) \biggr)\rightarrow0 \quad\text{as }n\rightarrow\infty.\end{aligned} $$

It follows that for all *n* large enough,

$$ t^{-1} \Biggl\vert \sum_{i=1}^{n}a_{ni} \hat{\mathbb{E}}Z_{it} \Biggr\vert < 1/4, $$

which implies that

$$ H_{2}\leq C \sum_{n=1}^{\infty}n^{\alpha p-2}l(n) \int_{n^{\alpha}}^{\infty}\mathbb{V} \Biggl(t^{-1}\sum _{i=1}^{n}a_{ni}(Z_{ti}- \hat{\mathbb {E}}Z_{ti})>1/4 \Biggr)\,\text{d}t. $$

For fixed $t>n^{\alpha}$ and $n\geq1$, it is easily seen that $\{ a_{ni}(Z_{ti}-\hat{\mathbb{E}}Z_{ti}),i\geq1\}$ are still END random variables. Hence, we have by Markov’s inequality, Lemma 2.3, (4.3), (4.12), (4.13), Lemma 2.2(i) that

$$\begin{aligned} H_{2} \leq{}& C \sum_{n=1}^{\infty}n^{\alpha p-2-\alpha}l(n) \int _{n^{\alpha}}^{\infty}t^{-2}\sum _{i=1}^{n}a_{ni}^{2}\hat{\mathbb {E}}Z_{ti}^{2}\,\text{d}t \\ \leq{}& C \sum_{n=1}^{\infty}n^{\alpha p-1-\alpha}l(n) \int_{n^{\alpha}}^{\infty}t^{-2}\hat{ \mathbb{E}}X^{2}g \biggl(\frac{\mu X }{t} \biggr)\,\text{d}t \\ &+ C\sum_{n=1}^{\infty}n^{\alpha p-1-\alpha}l(n) \int_{n^{\alpha}}^{\infty}\hat{\mathbb{E}} \biggl(1-g \biggl( \frac{X}{t} \biggr) \biggr)\,\text{d}t \\ \leq{}&C\sum_{n=1}^{\infty}n^{\alpha p-1-\alpha}l(n)\sum_{m=n}^{\infty}\int_{m^{\alpha}}^{(m+1)^{\alpha}}t^{-2}\hat{\mathbb {E}}X^{2}g \biggl(\frac{ \mu X }{t} \biggr)\,\text{d}t \\ \leq{}&C\sum_{n=1}^{\infty}n^{\alpha p-1-\alpha}l(n)\sum_{m=n}^{\infty}m^{\alpha-1-2\alpha}\hat{\mathbb{E}}X^{2}g \biggl(\frac{\mu X }{(m+1)^{\alpha}} \biggr)\,\text{d}t \\ ={}&C\sum_{m=1}^{\infty}m^{\alpha-1-2\alpha} \hat{\mathbb {E}}X^{2}g \biggl(\frac{\mu X }{(m+1)^{\alpha}} \biggr)\sum _{n=1}^{m} n^{\alpha p-1-\alpha}l(n) \\ \leq{}&C\sum_{m=1}^{\infty}m^{\alpha-1-2\alpha}\hat{\mathbb {E}}X^{2}g \biggl(\frac{\mu X }{(m+1)^{\alpha}} \biggr)m^{\alpha p-\alpha }l(m) \\ ={}&C\sum_{n=1}^{\infty}n^{\alpha p-1-2\alpha}l(n)\hat{\mathbb {E}}X^{2}g \biggl(\frac{\mu X }{(n+1)^{\alpha}} \biggr) \\ \leq{}&C\sum_{n=1}^{\infty}n^{\alpha p-1}l(n) \mathbb{V}\bigl(|X|>\mu n^{\alpha}\bigr)< \infty.\end{aligned} $$

Hence, this finishes the proof of Theorem 3.2. □

### Proof of Theorem 3.3

We use the same notations as those in Theorem 3.1. The proof is similar to that of Theorem 3.1. We only need to show that

$$ n^{-\alpha} \Biggl\vert \sum_{i=1}^{n} \hat{\mathbb{E}}a_{ni}X_{i}^{(n)} \Biggr\vert \rightarrow0 \quad\text{as } n\rightarrow\infty. $$

Because $l(x)>0$ is a monotone nondecreasing function, we have

$$\begin{aligned} |X|^{1/\alpha} &= |X|^{1/\alpha} I\bigl(|X|\leq1\bigr)+|X|^{1/\alpha }l \bigl(|X|^{1/\alpha}\bigr)\frac{1}{l(|X|^{1/\alpha})}I\bigl(|X|>1\bigr) \\ &\leq 1+|X|^{1/\alpha}l\bigl(|X|^{1/\alpha}\bigr)\frac{1}{l(1)},\end{aligned} $$

which together with (3.8) yields that $C_{\mathbb{V}}|X|^{1/\alpha }< C_{\mathbb{V}}[|X|^{1/\alpha}l(|X|^{1/\alpha})]<\infty$. Noting that $1\leq1/\alpha<2$ and $\hat{\mathbb{E}}X_{i}=0$, we have

$$\begin{aligned} n^{-\alpha} \Biggl\vert \sum _{i=1}^{n}\hat{\mathbb {E}}a_{ni}X_{i}^{(n)} \Biggr\vert &\leq n^{-\alpha}\sum_{i=1}^{n}a_{ni} \bigl\vert \hat{\mathbb{E}}X_{i}-\hat{\mathbb{E}}X_{i}^{(n)} \bigr\vert \\ &\leq n^{-\alpha}\sum_{i=1}^{n}a_{ni} \hat{\mathbb{E}} \bigl\vert X_{i}-X_{i}^{(n)} \bigr\vert \\ &\leq Cn^{1-\alpha}\hat{\mathbb{E}}|X| \biggl(1-g \biggl( \frac {X}{n^{\alpha}} \biggr) \biggr) \\ &\leq Cn^{1-\alpha}\frac{\hat{\mathbb{E}}|X||X|^{1/\alpha -1}}{n^{1-\alpha}} \biggl(1-g \biggl( \frac{X}{n^{\alpha}} \biggr) \biggr) \\ &\ll C_{\mathbb{V}}\bigl(|X|^{1/\alpha}I\bigl(|X|>\mu n^{\alpha}\bigr)\bigr)\rightarrow0 \quad \text{as } n\rightarrow\infty.\end{aligned} $$

 □
